# Impact of overlapping risks of type 2 diabetes and obesity on coronavirus disease severity in the United States

**DOI:** 10.1038/s41598-021-96720-x

**Published:** 2021-09-09

**Authors:** Wataru Ando, Takeshi Horii, Takayuki Uematsu, Hideaki Hanaki, Koichiro Atsuda, Katsuya Otori

**Affiliations:** 1 Department of Clinical Pharmacy, Center for Clinical Pharmacy and Sciences, Kitasato University School of Pharmacy, 5-9-1 Shirokane, Minato-ku, Tokyo, 108-8641 Japan; 2 Laboratory of Pharmacy Practice and Science 1, Division of Clinical Pharmacy, Research and Education Center for Clinical Pharmacy, Kitasato University School of Pharmacy, Sagamihara City, Kanagawa 252-0375 Japan; 3 Biomedical Laboratory, Division of Biomedical Research, Kitasato University Medical Center, 6-100 Arai, Kitamoto City, Saitama 364-8501 Japan; 4 Infection Control Research Center, Ōmura Satoshi Memorial Institute, Kitasato University, 5-9-1 Shirokane, Minato-Ku, Tokyo, 108-8641 Japan

**Keywords:** Diabetes complications, Diabetes, Metabolic syndrome, Obesity, Risk factors, Outcomes research

## Abstract

The impact of overlapping risk factors on coronavirus disease (COVID-19) severity is unclear. To evaluate the impact of type 2 diabetes (T2D) and obesity on COVID-19 severity, we conducted a cohort study with 28,095 anonymized COVID-19 patients using data from the COVID-19 Research Database from January 1, 2020 to November 30, 2020. The mean age was 50.8 ± 17.5 years, and 11,802 (42%) patients were male. Data on age, race, sex, T2D complications, antidiabetic medication prescription, and body mass index ≥ 30 kg/m^2^ (obesity) were analysed using Cox proportional hazard models, with hospitalization risk and critical care within 30 days of COVID-19 diagnosis as the main outcomes. The risk scores were 0–4 for age ≥ 65 years, male sex, T2D, and obesity. Among the participants, 11,294 (61.9%) had obesity, and 4445 (15.8%) had T2D. T2D, obesity, and male sex were significantly associated with COVID-19 hospitalization risk. Regarding hospitalization risk scores, compared with those for hospitalization risk score 0 and critical care risk score 0, hazard ratios [95% confidence intervals] were 19.034 [10.470–34.600] and 55.803 [12.761–244.015] (*P* < 0.001) (*P* < 0.001), respectively, for risk score 4. Complications from diabetes and obesity increased hospitalization and critical care risks for COVID-19 patients.

## Introduction

Coronavirus disease (COVID-19) is currently one of the most concerning infections globally^1^. It has a high incidence and mortality rate in the United States^2^. The risk of increasing COVID-19 severity has been reported, with men having a 2.76-fold higher risk of hospitalization owing to COVID-19 than women. According to Grasselli et al.^3^, 82% of patients admitted to intensive care units (ICUs) were men. Obesity was reported to increase the risk of COVID-19, ICU admission, and death by 1.46-, 2.13-, and 1.74-fold, respectively, suggesting that obesity may affect lung function and immune function^4^. Furthermore, the greatest risk of severe COVID-19 is age^1^. Additionally, statistical analyses by the Center for Disease Control reported an increase in COVID-19 risk with an increase in age; compared with the 18–29-year-old group, the 85-year-old group had a 13- and 630-fold increase in hospitalization and mortality risks, respectively. Therefore, patient characteristics, such as male sex, obesity, and advanced age, can be risk factors for severe disease^5^. Studies reported that COVID-19 comorbidities, including hypertension, chronic heart disease, lung disease^6^, and type 2 diabetes (T2D), remarkably affected disease severity^7^.

T2D is a serious social problem in the United States^8^ because the number of newly diagnosed patients with T2D increased by 75% for all age groups between 1988 and 2010^9^. Moreover, less than half of all American adults do not follow the recommended guidelines for diabetes care^10^, and obese men aged > 60 years are more likely to develop T2D^11,12^. Therefore, risk factors for T2D are considered similar to those for severe COVID-19. Although several reports exist on the risk of severe COVID-19, studies on severe COVID-19 owing to overlapping risk factors are insufficient^13,14^. Thus, this study aimed to assess the impact of overlapping risk factors on COVID-19 severity using a large electronic medical record (EHR) database in the United States.

## Results

### Clinical characteristics

Among 28,093 COVID-19 patients, 11,802 (42%) were male, and 16,291 (58%) were female. The mean age was 50.8 ± 17.5 years. The number of hospitalized patients and those who received critical care services were 1075 (3.8%) and 336 (1.2%), respectively. The number of patients according to race were 20,078 Whites (71%), 7715 Blacks (28%), and 300 Asians (1%), and other interracial data are described in Supplementary Table S1. In total, 4445 (15.8%) patients had T2D. Glycated haemoglobin (HbA1c) information was available for 3498 (12.5%) patients: 2612 (74.7%) patients had HbA1c < 7% and 886 (25.3%) had HbA1c ≥ 7%. The most common drug administered was metformin, followed by incretin mimetic, sulfonylurea, SGLT2 inhibitor, and thiazolidinedione. Fewer patients received α-glucosidase and DPP4 inhibitors, insulin, and meglitinide. The body mass index (BMI) of 18,253 patients were determined: 6959 (38.1%) had BMI < 30 kg/m^2^, and 11,294 (61.9%) had BMI ≥ 30 kg/m^2^. The number of patients with fatty liver was 429 (1.5%).

### Analysis of severity factors of COVID-19

**Figure 1:**
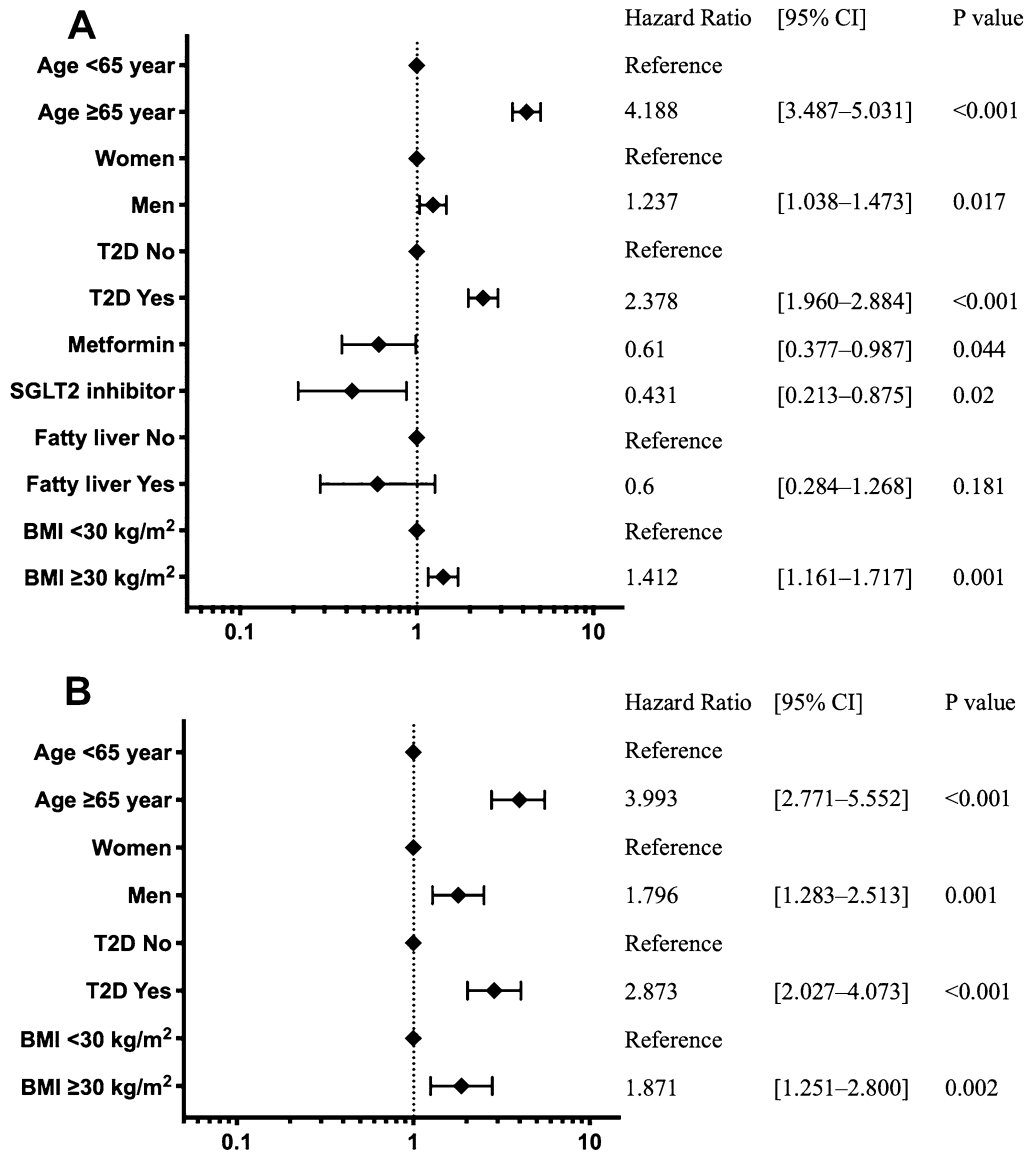
Risk analysis of hospitalization and critical care factors for COVID-19 using the Cox proportional hazards model. The forest plot indicates the HRs (diamonds) and 95% CIs (horizontal bars) for hospitalization risk (**A**) and critical care (**B**). The number of patients included (n = 28,093), those with BMI (n = 18,253), and those with HbA1c (n = 3498) for each analysis. BMI, body mass index; CI, confidence interval; SGLT2, sodium-glucose cotransporter 2; HR, hazard ratio; HbA1c; glycated haemoglobin.

The characteristics of hospitalized and critical care patients are shown in Table 1. Figure 1 depicts the risk of hospitalization and critical care within 30 days of COVID-19 diagnosis according to the multivariable Cox proportional hazards model analysis.

**Table 1:** Characteristics of patients with COVID-19 in hospitalization and critical care.

	No hospitalization	Hospitalization	*P* value	Critical care	*P* value
Number of patients, n (%)	n = 27,018	n = 1075		n = 336	
Age, mean (SD), years	50.2 (17.3)	65.5 (15.2)	< 0.001	65.5 (13.6)	0.9423
Sex (male/female)	11,238/15,780	564/511	< 0.001	206/130	0.004
T2D, n (%)	4094 (15)	351 (33)	< 0.001	123 (37)	0.18
HbA1c < 7%, n (%)	2569 (9)	43 (4)	0.309	17 (5)	0.656
HbA1c ≥ 7%, n (%)	852 (3)	34 (3)	0.309	11 (3)	0.656
Alpha-glucosidase inhibitor, n (%)	2 (0)	0 (0)	0.778	0 (0)	1
DPP4 inhibitor, n (%)	4 (0)	0 (0)	0.69	0 (0)	1
Incretin mimetic, n (%)	495 (2)	16 (1)	0.408	9 (3)	0.149
Insulin, n (%)	13 (0)	0 (0)	0.472	0 (0)	1
Meglitinide, n (%)	4 (2)	0 (0)	0.69	0 (0)	1
Metformin, n (%)	644 (2)	19 (2)	0.133	5 (1)	0.814
SGLT2 inhibitor, n (%)	379 (1)	10 (1)	0.194	4 (1)	0.674
Sulfonylurea, n (%)	392 (1)	13 (1)	0.508	4 (1)	0.978
Thiazolidinedione, n (%)	89 (0)	3 (0)	0.777	1 (0)	0.955
Fatty liver, n (%)	421 (2)	8 (1)	0.033	1 (0)	0.369
BMI mean (SD), kg/m^2^	33.4 (8.7)	35.5 (10.4)	< 0.001	36.4 (10.3)	< 0.001
BMI < 30 kg/m^2^, n (%)	6810 (25)	149 (14)	< 0.001	32 (10)	< 0.001
BMI ≥ 30 kg/m^2^, n (%)	10,938 (40)	356 (33)	< 0.001	108 (32)	< 0.001

Hospitalization (n = 1075) included critical care patients (n = 336). P value compared with the not-hospitalized group.

### Risks of hospitalization

Figure 1A shows the risk of hospitalization. T2D was significantly associated with an increased risk of hospitalization (Hazard ratio; HR 2.378, 95% Confidence interval [CI] {1.960–2.884}, *P* < 0.001). HRs for the risk of age ≥ 65 years and BMI ≥ 30 kg/m^2^ were 4.188 [3.487–5.031] (*P* < 0.001) and 1.412 [1.161–1.717] (*P* = 0.001), respectively. Administration of metformin and SGLT2 inhibitors significantly reduced hospitalization risk (HR 0.61 [0.377–0.987], *P* = 0.044 and HR 0.431 [0.213–0.875] *P* = 0.02, respectively). Additionally, males had higher risks of hospitalization than females (HR 1.237 [1.038–1.473], *P* = 0.017).

T2D, age, male sex, and obesity increased the risk of critical care (Fig. 1B).

### Risk of critical care

Critical care risk was mostly increased by T2D (HR 2.873, 95% CI [2.027–4.073], *P* < 0.001). Conversely, SGLT2 inhibitors, which reduced hospitalization risk, did not reduce critical care risk according to the univariate analysis (Supplementary Table S2). The presence of fatty liver did not affect the risk of hospitalization or critical care.

In the first half of 2020 (from January 1, 2020 to May 31, 2020 [early 2020]), 4136 patients were diagnosed with COVID-19. Of these patients, 257 (6.2%) were hospitalized and 70 (0.3%) were in critical care. In the second half (from June 1, 2020 to December 31, 2020 [late 2020]), 818 (3.4%) patients were hospitalized and 266 (1.1%) were in critical care. There was a significant increase in the number of patients admitted and those provided critical care in the second half compared with that in the first half of 2020 (*P* < 0.002, *P* = 0.001, respectively). Cox proportional hazards analysis of the number of hospitalized and critical care patients in early and late 2020 showed a similar trend in HRs (Supplementary Figure S1).

### Type 2 diabetes and risk with respect to race

Hospitalization risk according to race is presented in Table 2. T2D significantly increased hospitalization risk in Whites and Blacks (HR 2.185 [1.717–2.782] *P* < 0.001 and HR 2.447 [1.759–3.403], *P* < 0.001, respectively). In contrast, T2D did not affect hospitalization risk among Asians (HR 1.313 [0.212–0.770], *P* = 0.770). Increasing age significantly increased hospitalization risks in the three races, while BMI ≥ 30 kg/m^2^ increased the hospitalization risk in Whites (HR 1.538 [1.210–1.954], *P* < 0.001). Male patients had a significantly higher hospitalization risk only in the Black race (HR 1.551 [1.136–2.118], *P* = 0.006). Metformin administration reduced hospitalization risk in Whites (HR 0.546 [0.295–1.010], *P* = 0.054).

**Table 2:** Cox proportional hazards model for hospitalization and critical care risk factors of COVID-19 by race.

	White n = 20,078			Black n = 7715			Asian n = 300		
	HR	95% CI	*P* value	HR	95% CI	*P* value	HR	95% CI	*P* value
**Risk of hospitalization**									
Age < 65 years	1			1			1		
Age ≥ 65 years	5.244	4.165–6.601	< 0.001	2.7686	1.996–3.839	< 0.001	11.746	2.176–0.004	0.004
Female sex	1			1			1		
Male sex	1.167	0.941–1.447	0.159	1.551	1.136–2.118	0.006	0.993	0.22–0.993	0.993
T2D No	1			1			1		
T2D Yes	2.185	1.717–2.782	< 0.001	2.447	1.759–3.403	< 0.001	1.313	0.212–0.770	0.770
Metformin	0.546	0.295–1.010	0.054	0.609	0.246–1.506	0.283	2.1912	0.339–0.410	0.410
SGLT2 inhibitor	0.713	0.348–1.459	0.354	1			1		
Fatty liver No	1			1			1		
Fatty liver Yes	0.710	0.315–1.600	0.407	0.437	0.006–3.125	0.410	1		
BMI < 30 kg/m^2^	1			1			1		
BMI ≥ 30 kg/m^2^	1.538	1.210–1.954	< 0.001	1.179	0.822–1.691	0.371	0.35	0.042–0.332	0.332
**Risk of critical care**									
Age < 65 years	1			1			1		
Age ≥ 65 years	5.500	3.435–8.805	< 0.001	2.695	1.518–4.786	0.001	6.119	0.911–41.084	0.062
Female sex	1			1			1		
Male sex	1.502	0.972–2.321	0.067	2.873	1.644–5.019	< 0.001	1.71	0.274–10.654	0.566
T2D No	1			1			1		
T2D Yes	2.799	1.779–4.403	< 0.001	2.485	1.407–4.390	0.002	1.735	0.253–11.910	0.575
BMI < 30 kg/m^2^	1			1			1		
BMI ≥ 30 kg/m^2^	2.008	1.192–3.385	0.009	2.118	1.013–4.429	0.046	0.566	0.061–5.251	0.616

Risk analysis of hospitalization and critical care factors by race, using Cox proportional hazards model, HR = 1 as reference.

*BMI* body mass index, *SGLT2* sodium-glucose cotransporter 2.

### Differences in risk of hospitalization and critical care among racial groups

Higher hospitalization risk was observed in Blacks and Asians (HR 1.698 [1.386–2.032], *P* < 0.001 and 2.098 [1.036–4.246], *P* = 0.039, respectively) than in Whites. A similar trend was observed for critical care risk (Blacks, HR 2.118 [1.486–3.018], *P* < 0.001 and Asians 5.46 [2.185–13.624]).

### Risk scores for hospitalization and critical care

**Figure 2:**
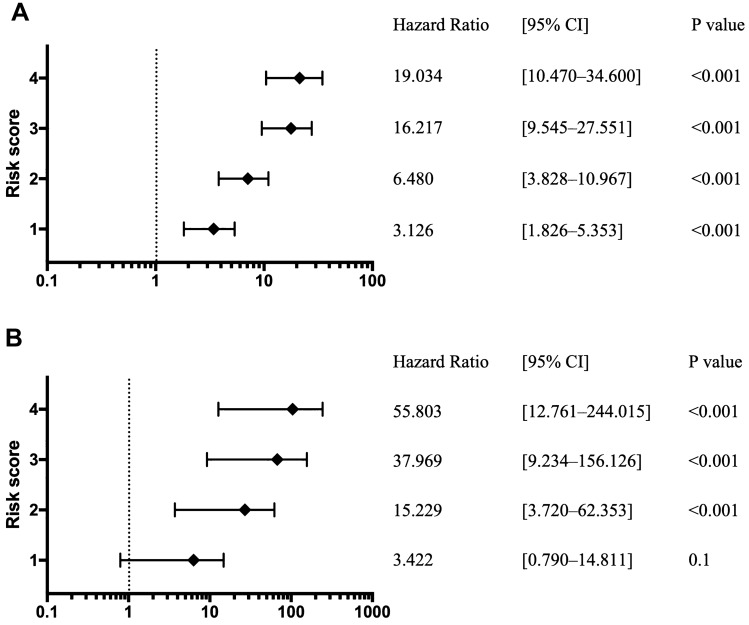
Relationship between increased risk factors and hospitalization and critical care due to COVID-19. The score calculation includes age ≥ 65 years, male sex, diabetes, and obesity with a BMI of ≥ 30 kg/m^2^, with each one scoring one point and resulting in a maximum of four points. The forest plot indicates the HRs (diamonds) and 95% CIs (horizontal bars) for risk of hospitalization (**A**) and critical care (**B**). HR, hazard ration; CI, confidence interval.

The hospitalization and critical care risk scores increased with T2D complications, age ≥ 65 years, high BMI, and male sex. Compared with those for hospitalization risk score 0, HRs and 95% CIs for hospitalization risk scores were 3.126 [1.826–5.353] (*P* < 0.001) for risk score 1 and 19.034 [10.470–34.600] (*P* < 0.001) for risk score 4 (Fig. 2A). Compared with those for critical care risk score 0, HRs and 95% CIs for critical care risk scores were 15.229 [3.72–62.353] (*P* < 0.001) for risk score 2, 37.969 [9.234–156.126] (*P* < 0.001) for risk score 3, and 55.803 [12.761–244.015] (*P* < 0.001) for risk score 4 (Fig. 2B). The risk of hospitalization and critical care for two combinations of the four risk factors with a risk score of 2 is shown in Supplementary Table S3, whereas Supplementary Table S4 describes that for three combinations of the four risk factors with a risk score of 3. In examining combinations of two or three of the four risk factors, age ≥ 65 years tended to most significantly increase the HR.

## Discussion

Our results indicated that T2D comorbidity, increasing age, male sex, and BMI ≥ 30 kg/m^2^ were associated with an increased hospitalization risk for COVID-19 patients in the United States. Hospitalization and critical care risks were markedly increased when two or more of the risk factors identified in the study were present. The combination of diabetes and metabolic syndrome may significantly increase infection risk^15,16^. In the United Kingdom, Hamer et al. reported that increased BMI was associated with the risk of COVID-19 hospitalization; additionally, controlling high-density lipoprotein and HbA1c levels reduced the risk of infection, and waist-to-hip ratio was associated with infection risk^17^. Thus, it has been suggested that glucose and lipid metabolism may be involved in the mechanism underlying increased hospitalization risk in obese patients.

Interestingly, although metformin and SGLT2 inhibitors reduced hospitalization risk in Whites, the risk was increased in patients with T2D. However, in Blacks and Asians, no risk reduction was observed with these drugs, possibly because of the lower number of prescriptions for metformin and SGLT2 inhibitors in Blacks and fewer Asian patients. Goodman et al. reported a decreased hospitalization risk and severe illness with diabetes medications in patients with COVID-19^16^. Moreover, Lalau et al. reported a prognostic analysis of patients with diabetes hospitalized for COVID-19; metformin reduced the odds of death for up to 28 days after hospitalization in metformin users compared with that in non-users^18^. Additionally, a meta-analysis of early reports of the COVID-19 epidemic revealed that metformin usage reduced mortality in the United States, China, and France^19^.

In the present study, metformin could reduce the severity of the disease in patients with diabetes, as it was observed to reduce hospitalization risk up to 30 days after diagnosis for patients using metformin. However, our results indicated that complications of diabetes were a major risk and should be seriously considered. The use of SGLT2 inhibitors in COVID-19 has been suggested to reduce the risk of cardiovascular events^20^. The anti-inflammatory properties of SGLT2 inhibitors may also be beneficial for patients with COVID-19 because they reduce the chance of cytokine storms^21^. Bossi et al.^22^ reported that SGLT2 inhibitors are associated with reduced T2D-related cardiovascular disease and mortality risks in COVID-19 patients, in addition to their hypoglycaemic effects^23^. The relationship between COVID-19 and SGLT2 inhibitors is currently being investigated in the DARE-19 study (NCT04350593)^24^. Conversely, some reports have suggested that the home administration of hypoglycaemic drugs does not affect mortality or adverse outcomes^25^. Therefore, the relationship between COVID-19 and diabetic drugs needs to be further assessed.

Older age was strongly associated with COVID-19 severity. However, glucose levels > 215 mg/dL and HbA1c > 9.5% on admission may be significantly associated with increased mortality in young adults with COVID-19^15^. Hence, diabetes increases the risk of death and severe illness irrespective of age. Other risk score studies of hospitalization due to COVID-19 have used a development cohort of 1590 patients and a validation cohort of 710 patients to assess risk factors; abnormal chest radiography, age, haemoptysis, dyspnoea, unconsciousness, number of comorbidities, history of cancer, neutrophil-to-lymphocyte ratio, lactate dehydrogenase, and direct bilirubin were included^26^. In a study of 11,721 cases across the United States, ADDM et al. reported that men, older age, obesity, geographic characteristics, chronic kidney disease, and pre-existing cardiovascular disease were associated with increased odds of needing mechanical ventilation^13^. The present study does not mention regionality. In a study of 3471 Americans, the regional characteristics of patients with COVID-19 lung lesions were reported to be higher in the South (81.6%) than in the Midwest (16.8%), and 55% were African American^14^. These reports strongly support our study findings. Therefore, the overlap of multiple risks, as identified by this study, may contribute to COVID-19 severity. Lowering the risk score by controlling T2D and obesity may reduce COVID-19 severity. Future studies are expected to include dyslipidaemia, cardiovascular disease, and anticoagulant use in risk analysis.

The proportion of patients hospitalized for COVID-19 was low, and the risk of critical care was even lower. Analysis for race revealed a higher risk of hospitalization among Blacks and Asians than among Whites^27–29^. However, the exact causes have not been identified. Complex environmental factors, such as genetic and social activity factors, may have an effect; hence, a more comprehensive evaluation is required. In an analysis of outcomes in a group of older adults receiving Medicaid in the United States, odds ratios for mortality outcomes were higher in the Black, Hispanic, and Native American groups than in the White group^30^. Conversely, economic disparities may have affected mortality and adverse outcomes^31^. The risks associated with race and background factors should be investigated carefully. As this study period was before the availability of SARS-CoV-2 vaccination, the relationship between potential risk and vaccination needs to be analysed in the future.

### Limitations

This study had some limitations. The Healthjump in the COVID-19 Research Database consists of EHRs extracted from a limited number of medical institutions of the United States. Not all patients with COVID-19 and diabetes have been represented, and HbA1c and BMI records could not be referenced for all patients. Therefore, further studies are required to evaluate the effects of blood glucose control and obesity on COVID-19.

The BMI data comprise values measured up to 6 months prior to this study and may not accurately reflect the values immediately before the diagnosis of COVID-19. Because of the small number of cases of fatty liver and the exclusive availability of Non-alcoholic fatty liver disease (NAFLD) in CPT4 codes, the risk of COVID-19 severity in patients with fatty liver should be studied in detail in a larger population. Confounding factors for hospitalization and critical care risks should consider non-diabetic complications such as cardiovascular events.

We hypothesized that there would be a period in the early phase of the epidemic (in the first half of 2020) when the high number of patients would overwhelm the health care system, and treatment of the most critically ill patients would be prioritized. Therefore, we attempted to examine the risk in early 2020. However, because of limited data in the early phase of the epidemic and concerns about accuracy, this database is not considered to have adequately accounted for the relative risks of outcomes related to diabetes, age, and BMI. Therefore, we hope that this issue will be clarified in the future as the risk of severe disease was unevenly distributed during the acute phase of the pandemic.

In examining the risk score, we considered the weight of the patients; however, it was difficult to set a reasonable criterion for age delimitation without considering background characteristics, such as race and lifestyle; therefore, we could not weigh the patients.

## Conclusions

COVID-19 patients who were both type-2 diabetics and obese had a significantly higher risk of post-diagnosis hospitalization and severe disease development. These findings suggest that efforts to manage diabetes and weight may reduce the severity of COVID-19.

## Methods

### Study design and data source

This study used the EHRs registered with the Healthjump database (Healthjump Inc., Philadelphia, PA, USA) and provided by the COVID-19 Research Database consortium (https://covid19researchdatabase.org). All personal information in this database was provided anonymously. Data were extracted by SQL using Snowflake (Snowflake Inc., San Mateo, CA, USA).

### Participants and definition

The Healthjump database, a large interoperable platform, receives and anonymizes the origin of EHR data over 500 healthcare organizations from across the United States. The data from January 1, 2020 to November 30, 2020 were extracted. Patients who met the following criteria were included in the study: (1) severe acute respiratory syndrome coronavirus 2 (SARS-CoV-2) DNA-positive patients (International Statistical Classification of Diseases and Related Health Problems, Tenth Revision; ICD-10: U07.1) and (2) age ≥ 20 years. In contrast, patients who met the following criteria were excluded: (1) missing information regarding race and sex, (2) suspected SARS-CoV-2 positivity (ICD-10: U07.2), and (3) type 1 diabetes. Data obtained from the EHR were age, sex, racial identity (White, Black, and Asian), 1-year antidiabetic medication history before SARS-CoV-2 diagnosis, HbA1c (NGSP), T2D (ICD-10: E11.65, E11.8, E11.9) or fatty liver diagnosis (K76.0), and BMIs of 6 months before the analysis. If more than one value was available for a measure, the value obtained closest to the diagnosis of COVID-19 was considered.

The Current Procedural Terminology 4th edition (CPT-4) and Healthcare Common Procedure Coding System (HCPCS) codes were referred to evaluate the rates of hospitalization and critical care. Patients with a code of critical care services (99,291–99,293 in CPT4 and HCPCS codes) were defined as critical care, those with Hospital Inpatient Care Services (99,221–99,223) were defined as hospitalization, and patients without either above code were defined as no hospitalization. For both diagnosis and CPT4 and HCPCS codes, the oldest code was used if the same code or the codes before and after were consecutive or overlapping. These codes represented COVID-19-related hospitalization from the date of diagnosis to the subsequent 30 days. The number of days to hospitalization and critical care services from the date of diagnosis was calculated and used in the Cox proportional hazards model.

The use of the following antidiabetic drugs was investigated in T2D patients: α-glucosidase inhibitor, dipeptidylpeptidase 4 (DPP4) inhibitor, incretin mimetic, insulin analogue, meglitinide, metformin, sodium-glucose cotransporter 2 (SGLT-2) inhibitor, sulfonylurea, and thiazolidinedione.

Patients with BMI ≥ 30 kg/m^2^ were classified as obesity^32^. Inpatient and critical care services were considered as the study outcomes, and T2D and its related factors were evaluated. Patients with BMI ≤ 15 kg/m^2^ or > 50 kg/m^2^ were excluded from this study.

### Calculation of hospitalization risk scores

Scores were calculated for items determined as significant in the hazard analysis, and the relationship between increasing values and hospitalization risk was analysed. For the determination of the risk score, all factors with HRs > 1 and P value of HRs < 0.05 in the multiple analysis were selected. The score was calculated by adding one point for each age ≥ 65 years, male sex, T2D, and BMI ≥ 30 kg/m^2^, and the relationship between the score and the risk of hospitalization and critical care were analysed. The scores ranged from 0 to 4.

### Missing data

In the case of insufficient BMI data, patients whose BMI data were entered but not in the range of ≤ 15 kg/m^2^ to > 50 kg/m^2^ were excluded from the statistical analysis for BMI. When data regarding antidiabetic medication were unavailable, we considered the case to have no prescription.

### Statistical analysis

Patient data following a normal distribution (age, BMI, HbA1c) are expressed as mean ± standard deviation values, and continuous variables were analysed using a one-tailed unpaired *t*-test. Categorical variables were analysed using a χ^2^ test and are expressed as absolute numbers and/or percentages. HRs for hospitalization or critical care risks were analysed using a Cox proportional hazards model (HR and 95% CI) that was adjusted for antidiabetic agents’ administration (α-glucosidase inhibitor, DPP4 inhibitor, incretin mimetic, insulin analogue, meglitinide, metformin, SGLT-2 inhibitor, sulfonylurea, thiazolidinedione, [yes/no]), sex, age (10-year increase), and BMI (cut-off 30 kg/m^2^). First, univariate analysis was used to extract factors with P value of HRs ≤ 0.2; thereafter, multivariate analysis was conducted using only those factors. The analyses of hospitalization and critical care risks for Whites, Blacks, and Asians were similarly performed. All statistical analyses were performed using STATA 16.0 Statistics for Windows (Stata Corp LLC, Texas, USA), and a *P* value less than 0.05 was considered statistically significant.

### Ethical considerations

This study was approved by the Ethical Committee of Kitasato University Hospital (No. 20-366). Because unlinked, anonymized data were used, the Ethics Committee confirmed that this study was not subject to compliance with the Ethical Guidelines for Medical and Health Research Involving Human Subjects and written informed consent was waived.

## Supplementary Information

Supplementary Information.

## Data Availability

The data that support the findings of this study are available from Healthjump database and the COVID-19 Research Database consortium but restrictions apply to the availability of these data, which were used under licence for the current study, and so are not publicly available. Data are however available from the authors upon reasonable request and with permission of Healthjump database and the COVID-19 Research Database consortium.
