# Supplementary material for: Impact of overlapping risks of type 2 diabetes and obesity on coronavirus disease severity in the United States
Source: Sci Rep. 2021 Sep 9;11:17968. doi: 10.1038/s41598-021-96720-x (PMC8429758; doi:10.1038/s41598-021-96720-x)
Supplement: Supplementary file 1 — Supplementary Information. [file 41598_2021_96720_MOESM1_ESM.docx]

**Supplementary information**

Impact of overlapping risks of diabetes and obesity on coronavirus disease severity in the United States

Wataru Ando, Ph.D.1*; Takeshi Horii, Ph.D.; Takayuki Uematsu, Ph.D.; Hideaki Hanaki, Ph.D.; Koichiro Atsuda, Ph.D.; Katsuya Otori, Ph.D.

* Corresponding author: Wataru Ando, PhD, Assistant Professor

Department of Clinical Pharmacy, Center for Clinical Pharmacy and Sciences, Kitasato University School of Pharmacy

5-9-1 Shirokane, Minato-ku, Tokyo 108-8641, Japan

E-mail: andow@pharm.kitasato-u.ac.jp

Number of Tables: 4

Number of Figures:1

Supplementary Table S1. Patient characteristics and background with respect to race

Supplementary Table S2. Risk analysis of hospitalization and critical care factors associated with COVID-19

Supplementary Table S3. Relationship of two risk factors combination in COVID-19 hospitalization and critical care

Supplementary Table S4. Relationship of three risk factors combination in COVID-19 hospitalization and critical care

Supplementary Figure S1. Risk analysis of hospitalization and critical care factors in early or late 2020 for COVID-19 using the Cox proportional hazards model

Supplementary Table S1. Patient characteristics and background with respect to race

| **Race** | **White n=20,078** | | **Black n=7,715** | | **Asian n=300** | | **Total n=28,093** | |
| --- | --- | --- | --- | --- | --- | --- | --- | --- |
| Age, mean (SD), year | 51.3 | (17.8) | 49.4 | (16.6) | 47.5 | (17.2) | 50.8 | (17.5) |
| Sex (male/female) | 8,890/11,188 |  | 2,770/4,945 |  | 142/158 |  | 11,802/16,291 | |
| Number of hospitalized patients, n(%) | 608 | (3.0) | 456 | (5.9) | 11 | (3.7) | 1,074 | (3.8) |
| Number of critical care patients, n(%) | 159 | (0.8) | 172 | (2.2) | 5 | (1.7) | 336 | (1.2) |
| T2DM, n(%) | 2,853 | (14.2) | 1,546 | (20.0) | 46 | (15.3) | 4,445 | (15.8) |
| Fatty liver, n(%) | 362 | (1.8) | 59 | (0.8) | 8 | (2.7) | 429 | (1.5) |
| α-GI, n(%) | 2 | (0) | 0 | (0) | 0 | (0) | 2 | (0) |
| DPP4 inhibitor, n(%) | 3 | (0) | 1 | (0) | 0 | (0) | 4 | (0) |
| Incretin mimetic, n(%) | 354 | (1.8) | 154 | (2.0) | 3 | (1.0) | 511 | (1.8) |
| Insulin, n(%) | 11 | (0.1) | 2 | (0) | 0 | (0) | 13 | (0) |
| Meglitinide, n(%) | 1 | (0) | 2 | (0) | 1 | (0.3) | 4 | (0) |
| Metformin, n(%) | 488 | (2.4) | 158 | (2.0) | 16 | (5.3) | 662 | (2.4) |
| SGLT2 inhibitor, n(%) | 272 | (1.4) | 110 | (1.4) | 7 | (2.3) | 389 | (1.4) |
| Sulfonylurea, n(%) | 267 | (1.3) | 133 | (1.7) | 5 | (1.7) | 405 | (1.4) |
| Thiazolidinedione, n(%) | 59 | (0.3) | 32 | (0.4) | 1 | (0.3) | 92 | (0.3) |
| BMI mean (SD), kg/m^2^ | 32.9 | (8.4) | 35.8 | (0.5) | 27.4 | (9.5) | 33.5 | (8.7) |
| BMI <30 kg/m^2^, n(%) | 5685 | (40.6) | 1,133 | (27.6) | 141 | (65.9) | 6,959 | (38.1) |
| BMI ≥30 kg/m^2^, n(%) | 8268 | (59.1) | 2,954 | (72.0) | 72 | (33.6) | 11,294 | (61.9) |
| HbA1c <7%, n(%) | 1886 | (76.1) | 681 | (70.9) | 45 | (75.0) | 2,612 | (74.7) |
| HbA1c ≥7%, n(%) | 591 | (23.9) | 280 | (29.1) | 15 | (25.0) | 886 | (25.3) |

BMI, body mass index; DPP4, dipeptidyl peptidase-4; α-GI, alpha-glucosidase inhibitor; SGLT2, sodium-glucose cotransporter 2, T2DM; type 2 diabetes.

Supplementary Table S2. Risk analysis of hospitalization and critical care factors associated with COVID-19

|  | Hospitalization | | | Critical care | | |
| --- | --- | --- | --- | --- | --- | --- |
|  | HR | 95% CI | P value | HR | 95% CI | P value |
| Age (10-year increase) | 1.669 | [1.606–1.734] | <0.001 | 1.656 | [1.546–1.774] | <0.001 |
| Age ≥65 year | 4.491 | [3.982–5.065] | <0.001 | 4.395 | [3.546–5.450] | <0.001 |
| Male sex | 1.530 | [1.358–1.725] | <0.001 | 2.194 | [1.762–2.734] | <0.001 |
| Female sex | 0.654 | [0.580–0.737] | <0.001 | 0.456 | [0.366–0.567] | <0.001 |
| T2D | 2.599 | [2.288–2.952] | <0.001 | 3.078 | [2.465–3.843] | <0.001 |
| HbA1c ≥7% | 2.335 | [1.489–3.662] | <0.001 | 1.897 | [0.889–4.051] | 0.098 |
| α-GI | 1 |  |  | 1 | — |  |
| DPP4 inhibitor | 1 |  |  | 1 | — |  |
| Incretin mimetic | 0.814 | [0.497–1.333] | 0.413 | 1.486 | [0.766–2.882] | 0.241 |
| Insulin | 1 | — |  | 1 | — |  |
| Meglitinide | 1 | — |  | 1 | — |  |
| Metformin | 0.708 | [0.444–1.228] | 0.146 | 0.63 | [0.260–1.523] | 0.305 |
| SGLT2 inhibitor | 0.667 | [0.358–1.244] | 0.203 | 0.859 | [0.320–2.301] | 0.762 |
| Sulfonylurea | 0.834 | [0.482–1.440] | 0.514 | 0.821 | [0.306–2.202] | 0.696 |
| Thiazolidinedione | 0.849 | [0.273–2.636] | 0.777 | 0.903 | [0.127–6.427] | 0.919 |
| Fatty liver | 0.481 | [0.240–0.964] | 0.039 | 0.191 | [0.027–1.362] | 0.099 |
| BMI <30 kg/m^2^ | 0.679 | [0.560–0.821] | <0.001 | 0.480 | [0.324–0.712] | <0.001 |
| BMI ≥30 kg/m^2^ | 1.475 | [1.219–1.786] | <0.001 | 2.083 | [1.404–3.091] | <0.001 |

Univariate analysis output were used for the Cox proportional hazards model.

This univariate analysis was used to extract factors with the P value of HRs less than 0.2, and multivariate analysis (Figure 1) was subsequently conducted using only those factors.

Number of patients included (n = 28,093), those with BMI (n = 18,253), and those with HbA1c (n = 3,498)

BMI, body mass index; DPP4, dipeptidyl peptidase-4; α-GI, alpha-glucosidase inhibitor; SGLT2, sodium-glucose cotransporter 2.

Supplementary Table S3. Relationship of two risk factors combination in COVID-19 hospitalization and critical care

| **Risk of administration** |  |  |  |
| --- | --- | --- | --- |
| Risk combinations | HR | 95% CI | P value |
| Male & age ≥65 years | 6.621 | [5.565–7.879] | <0.001 |
| Male & T2DM | 3.682 | [3.061–4.428] | <0.001 |
| Male & BMI ≥30 kg/m^2^ | 1.883 | [1.447–2.451] | <0.001 |
| Age ≥65 years & T2DM | 6.751 | [5.648–8.069] | <0.001 |
| T2DM & BMI ≥30 kg/m^2^ | 3.714 | [2.932–4.704] | <0.001 |
| Age ≥65 years & BMI ≥30 kg/m^2^ | 10.670 | [7.600–14.978] | <0.001 |
| **Risk of critical care** |  |  |  |
| Risk combinations | HR | 95% CI | P value |
| Male & age ≥65 years | 9.287 | [6.754–12.769] | <0.001 |
| Male & T2DM | 6.439 | [4.648–8.020] | <0.001 |
| Male & BMI ≥30 kg/m^2^ | 4.154 | [2.284–7.553] | <0.001 |
| Age ≥65 years & T2DM | 7.842 | [5.732–10.728] | <0.001 |
| T2DM & BMI ≥30 kg/m^2^ | 6.543 | [4.005–10.688] | <0.001 |
| Age ≥65 years & BMI ≥30 kg/m^2^ | 21.618 | [9.301–50.249] | <0.001 |

For score calculation, age over 65 years, male sex, type 2 diabetes (T2DM), and obesity (BMI ≥30 kg/m^2^) were assigned a point of one, respectively, and two were combined. The resulting maximum score was 2 points. Risk analysis of hospitalization and critical care factors was conducted using a Cox proportional hazards model.

Supplementary Table S4. Relationship of three risk factors combination in COVID-19 hospitalization and critical care

| **Risk of administration** |  |  |  |
| --- | --- | --- | --- |
| Risk combinations | HR | 95% CI | P value |
| Male & age ≥65 years & T2DM | 8.736 | [6.705–11.383] | <0.001 |
| Male & age ≥65 years & BMI ≥30 kg/m^2^ | 13.900 | [8.544–22.611] | <0.001 |
| Male & T2DM & BMI ≥30 kg/m^2^ | 4.645 | [3.329–6.481] | <0.001 |
| Age ≥65 years & T2DM & BMI ≥30 kg/m^2^ | 17.132 | [11.390–25.768] | <0.001 |
| **Risk of critical care** |  |  |  |
| Risk combinations | HR | 95% CI | P value |
| Male & age ≥65 years & T2DM | 14.718 | [9.210–23.519] | <0.001 |
| Male & age ≥65 years & BMI ≥30 kg/m^2^ | 22.382 | [7.850–63.810] | <0.001 |
| Male & T2DM & BMI ≥30 kg/m^2^ | 18.606 | [7.796–44.405] | <0.001 |
| Age ≥65 years & T2DM & BMI ≥30 kg/m^2^ | 58.185 | [17.757–190.6] | <0.001 |

For score calculation, age over 65 years, male sex, type 2 diabetes (T2DM), and obesity (with BMI ≥30 kg/m^2^) were assigned a point of one, respectively, and three were combined. The resulting maximum score was 2 points. Risk analysis of hospitalization and critical care factors was conducted using a Cox proportional hazards model.


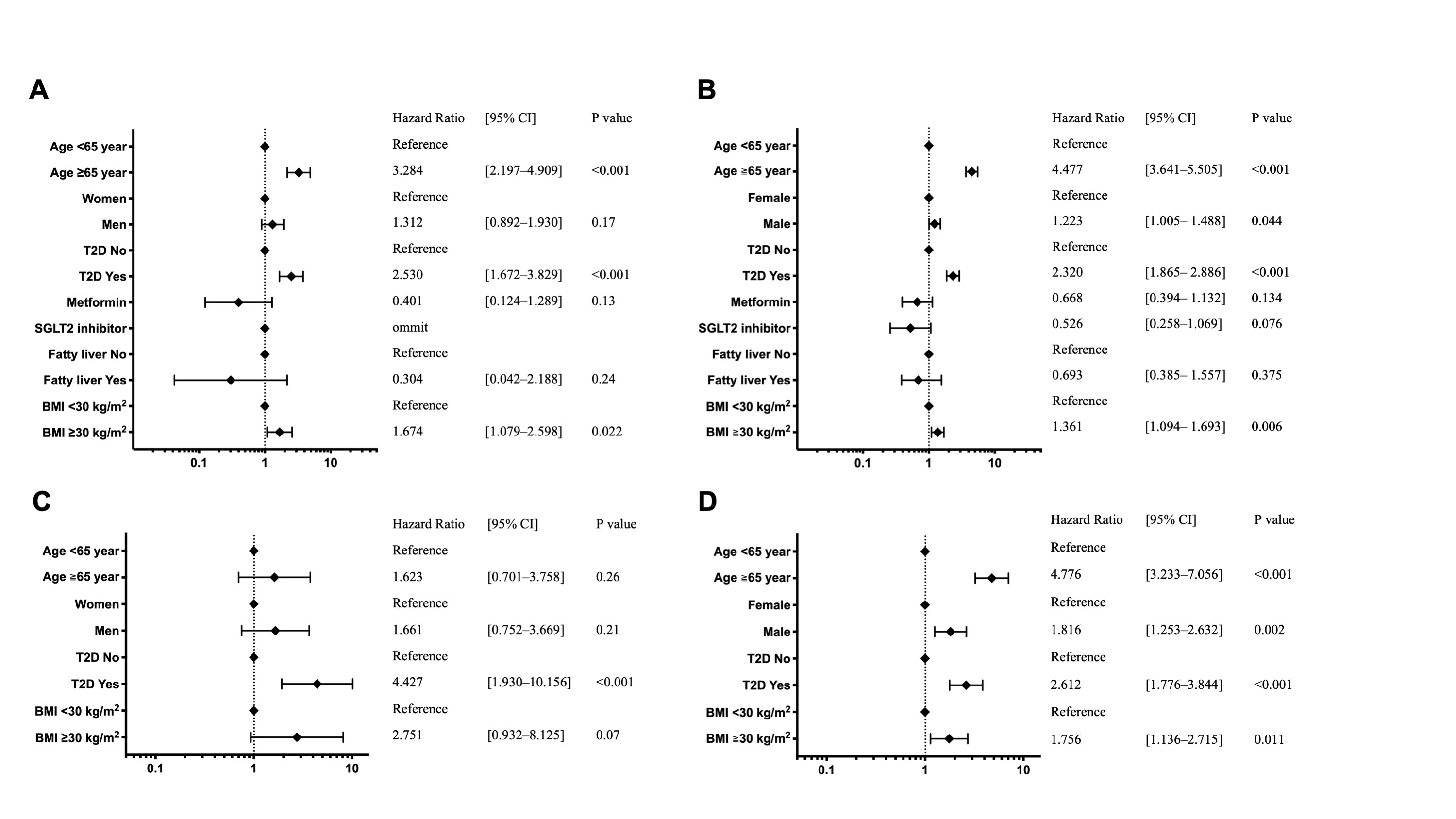
Supplementary Figure S1. Risk analysis of hospitalization and critical care factors in early or late 2020 for COVID-19 using a Cox proportional hazards model

The forest plot indicates the HRs (diamonds) and 95% CIs (horizontal bars) for hospitalization risk in early 2020 (A) and late 2020 (B) and critical care in early 2020 (C) and late 2020 (D). BMI, body mass index; CI, confidence interval; SGLT2, sodium-glucose cotransporter 2.
